# Trophic Mechanisms for Exercise-Induced Stress Resilience: Potential Role of Interactions between BDNF and Galanin

**DOI:** 10.3389/fpsyt.2014.00090

**Published:** 2014-07-28

**Authors:** Philip V. Holmes

**Affiliations:** ^1^Neuroscience Program, Psychology Department, Biomedical and Health Sciences Institute, The University of Georgia, Athens, GA, USA

**Keywords:** galanin, running, BDNF, resilience, psychological, anxiety, depression

## Abstract

Current concepts of the neurobiology of stress-related disorders, such as anxiety and depression emphasize disruptions in neural plasticity and neurotrophins. The potent trophic actions of exercise, therefore, represent not only an effective means for prevention and treatment of these disorders, they also afford the opportunity to employ exercise paradigms as a basic research tool to uncover the neurobiological mechanisms underlying these disorders. Novel approaches to studying stress-related disorders focus increasingly on trophic factor signaling in corticolimbic circuits that both mediate and regulate cognitive, behavioral, and physiological responses to deleterious stress. Recent evidence demonstrates that the neural plasticity supported by these trophic mechanisms is vital for establishing and maintaining resilience to stress. Therapeutic interventions that promote these mechanisms, be they pharmacological, behavioral, or environmental, may therefore prevent or reverse stress-related mental illness by enhancing resilience. The present paper will provide an overview of trophic mechanisms responsible for the enhancement of resilience by voluntary exercise with an emphasis on brain-derived neurotrophic factor, galanin, and interactions between these two trophic factors.

## Trophic Mechanisms for Exercise-Induced Stress Resilience: Potential Role of Interactions between BDNF and Galanin

Clinical studies convincingly establish the efficacy of exercise in the treatment of anxiety and affective disorders (1–3). However, understanding the neurobiological mechanisms responsible for these beneficial effects, a goal that necessitates the application of rodent models, remains a challenge. Since deleterious stress lies at the core of anxiety and depression, understanding the neurobiological impacts of stress, and the mechanisms for adapting to it will significantly enhance the development of more effective prevention and treatment. Recent advances in exercise neuroscience using rodent models have shed new light on the neural mechanisms by which physical activity produces long-term adaptations in brain circuits implicated in anxiety and depressive disorders. This literature reveals that the most significant impact of exercise on stress may not pertain to regulating transient states occurring in the presence of the stressor, but rather on moderating the long-term impact that acute stress may incur on subsequent stress events. The key benefit of exercise may thus involve the promotion of stress resilience.

The present paper will focus on stress resilience as a fundamental phenomenon underlying the beneficial effects of exercise, and it will briefly review the neurotrophic hypothesis of stress resilience. Two trophic mechanisms that may be involved in the stress resilience will be examined; one involving the widely studied brain-derived neurotrophic factor (BDNF) system and another based on the lesser-known trophic actions of the peptide galanin. The purpose of this comparison will be to point out a critical area for future investigation, which should aim to uncover how different trophic mechanisms may interact to promote optimal neural function.

## Effects of Exercise in Rodent Models of Depression and Anxiety and Their Implications for Stress Resilience

Though exercise represents an effective intervention for stress-related disorders in humans, evidence for its antidepressant and anxiolytic efficacy in rodent behavioral models is surprisingly inconclusive. In recent, comprehensive reviews of the anxiolytic and antidepressant effects of exercise in rodent models a common theme emerges, in which that exercise produces mixed and conflicting effects in standard rodent tests of anxiety and depression (4–6). Much of the conflicting evidence derives from the species or strain used as subjects, duration, and mode of exercise employed as the independent variable, and the selection of model that serves as the dependent measure of depressive- or anxiety-like behavior. Experiments employing forced modes of exercise, such as treadmill or swimming, introduce the confounding factor of stressor exposure on subsequent measures of stress-related behavior and are thus difficult to interpret. Furthermore, rodent paradigms that putatively model depressive-like behavior are plagued by questions of validity, and the most widely employed “models,” such as the forced swim or tail suspension tests, are better characterized as bioassays or screens for pharmacological manipulations with potential antidepressant activity (7). A particularly problematic aspect of these models is that they are too often assumed to represent the chronic, self-perpetuating nature of major depression by assessing a “snapshot” of spontaneous responsiveness to an acute stressor when no predisposing factors have been manipulated. They also assume that antidepressant actions may be observed in a healthy subject. It is with these caveats in mind that one must cautiously interpret the reports that exercise may exert either antidepressant-like, “pro-depressive,” or no effects in the forced swim or similar tests (5, 8, 9).

Similar problems of interpretation arise when critically examining exercise effects in anxiety models. When such models are applied as a single measure of spontaneous behavior in the presence of the mild stress associated with standard tests, it is difficult to ascertain whether increased responding reflects behavior reminiscent of anxiety or, rather, adaptive coping to the exigencies of the stressor. Both interpretations have been proposed in previous studies, and exercise has been variously characterized that has anxiogenic, anxiolytic, or without effect (4–6). This range of contradictory results is evident even when focusing on a single model, the elevated plus-maze. After systematically reviewing this literature, we have proposed that understanding the effects of exercise hinge upon examination of the longitudinal impact of stress exposure on subsequent responding (6). We have thus shown that the anxiolytic-like effects of exercise are only consistently observed in standard models of anxiety when rats have been previously exposed to a different type of stressor. Exercise, thus appears to promote resilience to stress.

## Trophic Mechanisms in Stress Resilience

Emerging concepts of the neurobiology of stress-related disorders, such as anxiety and depression emphasize disruptions in neural plasticity and trophic mechanisms (10, 11). Current research on the neurobiology of stress, thus, increasingly involves measures of neurogenesis, dendritic arborization, dendritic spine maintenance, synaptogenesis, and other forms of plasticity. This new perspective on the neurobiological basis of anxiety and depressive disorders is superseding traditional explanations that emphasize dysfunction in monoaminergic transmission as the primary etiological mechanism. The focus is thus shifting to the role of disruptions in trophic factor signaling, especially in corticolimbic circuits that mediate and/or regulate cognitive, behavioral, and physiological responses to stress. Recent evidence demonstrates that the neural plasticity supported by these trophic mechanisms is vital for establishing and maintaining stress resilience (12, 13). Stress-induced atrophy of the hippocampus has been linked to decreased resilience in clinical populations and rodent models (11, 14–16). Maintenance of plasticity in the medial prefrontal cortex (mPFC) also plays a crucial role in stress resilience, and disturbances in this plasticity are linked to the pathophysiology of depression and anxiety (17–21). The translational value of this new perspective on trophic mechanisms will be the identification and development of therapeutic interventions that enhance resilience-promoting plasticity, be they pharmacological, behavioral, or environmental.

## Role of Exercise-Induced Regulation of Hippocampal BDNF in Anxiety and Depression

The prominence of trophic mechanisms in determining the pathophysiology of anxiety and depression compels further examination of the profound influence exercise exerts on trophic factor expression. Much work already has demonstrated the capacity for exercise to induce a variety of trophic factors in the brain and periphery, such as BDNF, insulin-like growth factor (IGF-1), vascular endothelial growth factor (VEGF), and the inflammatory protein VGF (11), but most of this previous research has focused on BDNF signaling in the hippocampus (11, 22–24).

Brain-derived neurotrophic factor is a member of the neurotrophin family, a group of structurally related peptide growth factors that signal through TrkB receptors (22). BDNF is directly implicated in various forms of hippocampal plasticity induced by exercise (25, 26), leading many researchers to hypothesize that this action may mediate antidepressant and anxiolytic effects of exercise (11, 15, 23). This hypothesis is supported by numerous findings of relationships between exercise, BDNF, hippocampal plasticity, and antidepressant actions. For example, exercise consistently elevates BDNF expression in the hippocampus (23, 27, 28) and potentiates the antidepressant activity of antidepressant drugs in the forced swim test [(9); though c.f. (8)]. Antidepressant effects of exercise in mice are eliminated by BDNF knockout and inhibition of MAPK signaling, an intracellular mediator of BDNF (29). Exercise also reliably enhances hippocampal neurogenesis (30). This evidence has provided the foundation for the hypothesis that the beneficial effects of exercise are mediated specifically by BDNF-induced neurogenesis in the hippocampus. This hypothesis is an extension, what is generally referred to as the “neurogenesis hypothesis of affective disorders.” Some reports from the exercise literature support this hypothesis (11, 25), though systematic reviews of studies linking hippocampal BDNF to antidepressant actions in humans and rodent models found many disassociations between BDNF and depression (31, 32). With respect to neurogenesis specifically, exercise-induced hippocampal neurogenesis is associated with increased spontaneous anxiety-like behavior, an effect that is reversed after irradiation, which effectively eliminates neurogenesis (33, 34). As described above, the discrepancies in the literature may relate to whether the behavioral paradigm measures acute stress reactivity or the longitudinal impact of stress (i.e., resilience). They may also depend on the nature of trophic factor-mediated plasticity in the hippocampus. In addition to its neurogenic effects, exercise induces other forms of plasticity in hippocampal neurons, such as alterations in dendritic spine architecture (23), which must be considered in accounting for antidepressant or anxiolytic effects.

The studies presented above establish links between exercise, BDNF, and antidepressant actions. However, whether BDNF-dependent actions of exercise in the hippocampus reflect antidepressant effects *per se* or some underlying process such as stress resilience is not clear. Hippocampal administration of BDNF does not reverse the exaggerated stress response exhibited by rats previously exposed to uncontrollable stress, and a pharmacological manipulation that reduces hippocampal BDNF does not exacerbate the effects of stress on subsequent stress responses (35). These findings suggest that BDNF signaling in the hippocampus does not have a generalized influence on stress resilience.

## Potential Role of Exercise-Induced Regulation of Locus Coeruleus Galanin in Stress Resilience

As discussed above, exercise increases the expression of a wide range of trophic factors throughout the brain. A fuller understanding of how trophic mechanisms are involved in the beneficial effects of exercise on stress-related behaviors, therefore, requires expanding the scope beyond BDNF in the hippocampus and examining other trophic systems. Galanin is a neuromodulatory peptide and trophic factor that exerts multiple effects through its interaction with specific G protein-coupled receptor subtypes designated GalR1, GalR2, and GalR3 (36, 37). Both galanin and its receptors are widely distributed in several brain systems, and galanin signaling thus impacts a variety of cognitive, behavioral, and endocrine functions. Some of the highest concentrations of galanin are found in the locus coeruleus (LC), where galanin is colocalized in over 80% of noradrenergic neurons (38). Galanin-containing LC neurons extensively innervate the mPFC, where galanin-immunoreactive terminals and GalR2 receptors are present in relatively high densities (39–41).

Research from my laboratory has repeatedly shown that 3 weeks of voluntary exercise elevates galanin gene expression in the LC in a running distance-dependent fashion (42–44). We have also shown that exercise exerts antidepressant effects in chronic models of depression (45), and chronic antidepressant treatment elevates galanin mRNA in the LC similarly to exercise (46). This evidence raises the obvious question of whether galanin exerts antidepressant and/or anxiolytic effects. Reviews of the extensive literature on the effects of galanin on anxiety- and depression-related behaviors show that galanin’s effects on these behaviors, like those of exercise, are mixed and conflicting (47). Most of these previous experiments involved acute, central administration of galanin receptor ligands with subsequent tests of spontaneous behavior in standard models. With regard to galanin/GalR1/GalR2 transgenic mice, the findings are even more conflicting with predominantly negative findings in most standard anxiety and depression models (47).

Despite these confusing results, the behavioral evidence is clearer in experiments that examined galanin’s influence on the longitudinal impact of stress. For example Lu et al. (48) reported no effect of GalR2 knockout on spontaneous behaviors such as elevated plus-maze, light/dark transition, forced swim, or tail suspension tests. However, GalR2 knock-out mice exhibited increased susceptibility to the repeated stress employed in a “learned helplessness”-like paradigm compared to wild type mice. Unlike the other tests, “learned helplessness” paradigms examine the impact of uncontrollable stress exposure on subsequent responding to repeated stressors. Loss of GalR2 led to a susceptible phenotype in this paradigm, suggesting that galanin signaling through GalR2 is necessary for resilience. Further supporting a specific role for GalR2 in resilience, transgenic overexpression of GalR2 in several frontocortical areas, including mPFC, was found to decrease immobility in a version of the forced swim test that involved pre-exposure to swim stress on the previous day (49). In contrast, GalR2 overexpression had no effect on elevated plus-maze or novel open field exploration in mice not previously exposed to stress. The behavioral literature thus points to functions for galanin beyond neuromodulatory effects. Galanin and GalR2 in particular, are evidently involved in protecting against the lasting consequences of an acute stress event by diminishing reactivity to subsequent stressors rather than by modulating reactivity to the acute stress event itself. This long-term protection suggests a role for galanin in the plasticity underlying resilience.

Galanin is well positioned as a trophic factor expressed in high concentrations in the LC to modify neural architecture in stress-responsive targets such as the mPFC. As described above, maintenance of dendritic spines in the mPFC may provide a cellular mechanism for resilience. Recent studies of galanin have revealed specific actions on neurite dynamics mediated by activation of GalR2 (50, 51). GalR2-mediated activation of Gq/11 influences multiple downstream targets, which includes inhibition of the RhoA GTPase. Galanin thus promotes neurite formation in neuronal cultures through a pathway involving GalR2-mediated inhibition of RhoA-ROCK signaling through LIMK, with subsequent activation of the actin-binding protein cofilin (51). Stress is associated with excess activation of RhoA, which leads to a reduction in spine densities (52). GalR2-mediated inhibition of RhoA-ROCK signaling thus provides a hypothetical mechanism for galanin-mediated protection against stress-induced spine atrophy. GalR2-signaling may also stabilize neurites via maintenance of microtubule integrity by promoting aggregation of microtubule-associated protein 2 (MAP2) and β-tubulin (53), a process that may also involve inhibition of RhoA (54). These actions of galanin represent candidate mechanisms for protection against dendritic spine atrophy induced by stress.

## Potential Interactions between BDNF and Galanin in Optimizing Neuronal Function

Though BDNF and galanin share common neurotrophic functions, focusing on how the systems interact provides a new approach to understanding neural plasticity. Intracellular signaling of BDNF and galanin converges in many pathways, but their mutual influence on MAP2 may reflect complementary mechanisms to promote spine maintenance during the physiological challenges imposed by stress at the cellular level. TrkB-mediated BDNF signaling promotes the expression of MAP2 and microtubule assembly (54), whereas GalR2-signaling may be more involved in maintaining microtubule assemblies by inhibiting MAP2 phosphorylation, as described above. The two trophic systems may thus mutually maintain microtubule integrity, but through distinct mechanisms. Conversely, BDNF and galanin signaling pathways diverge in the area of coflin activation. Binding of BDNF to TrkB activates a LIMK through a RAC1 pathway, which ultimately leads to the inactivation of cofilin (54). That GalR2-signaling leads to the activation of cofilin through inhibition of LIMK suggests that BDNF and galanin may exert counter-regulatory influences in their mutual function to promote spine maintenance.

Counter–regulatory interactions between galanin and BDNF are also evident at the systems level. Left unchecked, exercise-induced elevations in BDNF may lead to state of neuronal hyperexcitability (22). Exercise induces a long-term enhancement of glutamatergic activity through upregulation of NMDA receptors (30). Additionally, enhanced transmission through AMPA receptors promotes Ca^++^-mediated BDNF release and signaling through TrkB (11, 54). The resulting BDNF-mediated effects on synaptic plasticity may further enhance excitatory glutamatergic transmission. This positive feedback loop between BDNF and glutamatergic activities accounts for the increased seizure vulnerability and excitotoxicity seen following experimental manipulations that enhance BDNF function, particularly in the hippocampus (55–57). This potential state of hyperexcitability following BDNF upregulation may also account for the finding of increased vulnerability to kainic acid-induced excitotoxicity following direct injection into the hippocampus of anesthetized rats that had undergone several weeks of exercise (58). In contrast to this finding in anesthetized rats, we have shown decreased vulnerability to kainic acid-induced seizures in awake, freely behaving rats (59). The galanin receptor antagonist M40 blocked this exercise-induced protection against seizures. Taken together, the evidence suggests that under normal physiological conditions, the potential state of hippocampal hyperexcitability induced by elevated BDNF may be regulated by the galanin system. The anticonvulsant and neuroprotective properties of both endogenous and exogenous galanin are well established (50, 60, 61). The dense innervation of the hippocampus by galaninergic projections originating from the LC (62), the presence of galanin receptors in this structure (63), and the increase in galanin with exercise (42–44), all point to this system as a regulatory mechanism controlling the deleterious consequences of exercise-induced upregulation of BDNF.

## Conclusion

The trophic influences of exercise, which are mediated by a wide range of neural and humoral factors, are well known. This body of knowledge fits well with new concepts of anxiety and depression as they relate to disturbances in neuroplasticity, and it reveals a compelling neurobiological mechanism that explains the beneficial effects of exercise on stress-related disorders. The literature from rodent models suggests that the primary benefit of exercise may be the promotion of resilience to stress. The translational value of this hypothesis may be realized through the promotion of exercise as a means to mitigate the longitudinal and cumulative impact of the repeated stressors. Exercise may thus afford both preventative and therapeutic benefits. Given the array of neurotrophins influenced by exercise, the next challenge for future research will be to examine how these varied mechanisms coordinate to optimize neuronal health. The interactions between BDNF and galanin represent an informative example of how trophic mechanisms that mutually promote the maintenance and survival of neurons may require reciprocal regulatory influences to achieve their ultimate benefit.

## Conflict of Interest Statement

The author declares that the research was conducted in the absence of any commercial or financial relationships that could be construed as a potential conflict of interest.
